# Perceptions of healthcare professionals on the use of a risk prediction model to inform atrial fibrillation screening: qualitative interview study in English primary care

**DOI:** 10.1136/bmjopen-2024-091675

**Published:** 2025-02-05

**Authors:** Ellen Hamilton, Lydia Shone, Catherine Reynolds, Jianhua Wu, Ramesh Nadarajah, Chris Gale

**Affiliations:** 1 Faculty of Medicine and Health, University of Leeds, Leeds, UK; 2 Leeds Institute of Cardiovascular and Metabolic Medicine, University of Leeds, Leeds, UK; 3 Queen Mary University of London, Queen Mary University of London, London, UK; 4 Department of Cardiology, Leeds Teaching Hospitals NHS Trust, Leeds, UK

**Keywords:** Primary Care, Electronic Health Records, Cardiovascular Disease

## Abstract

**Objectives:**

There is increasing interest in guiding atrial fibrillation (AF) screening by risk rather than age. The perceptions of healthcare professionals (HCPs) towards the implementation of risk prediction models to target AF screening are unknown. We aimed to explore HCP perceptions about using risk prediction models for this purpose, and how models could be implemented.

**Design:**

Semistructured interviews with HCPs engaged in the Future Innovations in Novel Detection of AF (FIND-AF) study. Data were thematically analysed and synthesised to understand barriers and facilitators to AF screening and guiding screening using risk assessment.

**Setting:**

Five primary care practices in England taking part in the FIND-AF study.

**Participants:**

15 HCPs (doctors, nurses/nurse practitioners, healthcare assistants, receptionists and practice managers).

**Results:**

Participants knew the health implications of AF and were supportive of the risk prediction models for AF screening. Four main themes developed: (1) health implications of AF, (2) positives and negatives of risk prediction in AF screening, (3) strategies to implement a risk prediction model and (4) barriers and facilitators to risk-guided AF screening. HCPs thought risk-guided AF screening would improve patient outcomes by reducing AF-related stroke, and this outweighed concerns over health anxiety and the impact on workload. Pop-up notifications and practice worklists were the main suggestions for risk-guided screening implementation and for this to be predominantly run by administrative staff. Many recommended the need for educating staff on AF and the prediction models to help aid the implementation of a clear protocol for longitudinal follow-up of high-risk patients and communication of risk.

**Conclusions:**

Overall, HCPs participating in the FIND-AF study were supportive of using risk prediction to guide AF screening and willing to take on extra workload to facilitate risk-guided AF screening. The best pathway design and the method of how risk is communicated to patients require further consideration.

**Trial registration number:**

NCT05898165.

**STRENGTHS AND LIMITATIONS OF THIS STUDY**

- Participants included different healthcare professionals (HCPs) working in primary care, both patient-facing and administrative, providing a holistic view of the impact of AF screening on primary care and the use of a risk prediction model within the process.
- General practice sites that were included in the interviews covered a range of socioeconomic deprivation levels.
- We report the perceptions of HCPs taking part in a study of AF screening targeted by a prediction model, which may impact the generalisability of our findings.

## Introduction

 Atrial fibrillation (AF) is the most common sustained cardiac arrhythmia worldwide and confers an increased risk of stroke, heart failure, cognitive decline and death.^1^ Moreover, it is estimated that up to 35% of disease burden remains undiagnosed^2^ and 15% of strokes occur in the context of undiagnosed AF.^3^ Early detection of AF may permit the initiation of oral anticoagulation to reduce embolic stroke risk.^4^ The European Society of Cardiology recommends opportunistic screening by pulse palpation or ECG rhythm strip in persons aged ≥65 years and systematic ECG screening in those aged ≥75 years or at elevated stroke risk.^1^

However, age-based AF screening is inefficient (a yield of only 3% in the STROKESTOP RCT)^2^ and excludes the increasing cohort of individuals aged younger than 65 years who are being diagnosed with AF and are eligible for anticoagulation.^5^ A large proportion of the population is registered in primary care with a routinely collected electronic health record (EHR),^6 7^ and primary care is frequently considered the optimum setting for AF screening.^8^ An algorithm that uses routinely collected primary care EHR data to calculate AF risk has been identified by AF screening stakeholders as a feasible and efficient approach to identify suitable individuals for screening.^8^ Recently, the British Heart Foundation sequentially funded the development and pilot implementation of the Future Innovations in Novel Detection of AF (FIND-AF) algorithm to stratify individual-level AF risk using primary care EHRs.^9^

Previous studies have investigated the views of the public on AF screening^10 11^ or the views of healthcare professionals (HCPs) and regulators on key propositions for successful delivery of AF screening programmes.^8 12^ The perceptions of general practitioners, nurse practitioners and healthcare assistants for the facilitators and barriers of AF screening have been explored^13 14^ but not those of the administrative staff and not specifically related to the implementation of a risk prediction model. We aimed to explore primary care HCPs’ perceptions about the barriers and facilitators to AF screening guided by AF risk prediction.

## Methods

This qualitative study was nested within the FIND-AF study.^15^ At 17 primary care sites in Yorkshire, England, individuals aged 30 years or older, without a history of AF and eligible for oral anticoagulation were recruited. From their primary care EHR data, participants were identified as higher risk and lower risk, and then a period of monitoring with a single-lead ECG device was conducted. Primary care sites identified eligible participants, sent out invitations and then received reports for whether the consented participants had been diagnosed with AF or not. Results are reported using the Standards for Reporting Qualitative Research.^16^

### Sample

The participating sites in the FIND-AF study were stratified by the Index of Multiple Deprivation, and the sites were invited to participate to represent an area from each stratum of socioeconomic classification, with all the sites accepting. We recruited a purposive sample of HCPs, including doctors, nurses/nurse practitioners, healthcare assistants, receptionists and practice managers,^17^ to give a holistic view of risk-guided AF screening. Email invitations explaining the aims of the study were sent to HCPs at each site and 15 agreed to participate.

### Data collection

Interviews were semistructured and used a flexible topic guide exploring barriers, facilitators and impacts of using a prediction model in AF screening in a primary care setting (online supplemental material). The topic guide was designed to reflect our research question and was refined and iteratively adapted as interviews progressed to accommodate areas of interest raised by the participants. Two researchers (EH and LS) conducted interviews by virtual teleconference. Consent was taken prior to each interview. The audio recorded interviews were carried out between April 2023 and January 2024 and lasted for an average 13 min each (range 8 to 20 min). We completed 15 interviews with 15 participants. Table 1 lists the participants’ characteristics.

**Table 1:** Demographics of the participants and recruitment sites

Participant characteristics	N=15
Role	
General practitioner	7
Nurse/nurse practitioner	4
Physician associate	1
Data administrative staff	2
Practice assistant manager	1
Age group	
18–30	3
31–50	10
50–60	2
60–70	0
70+	0
Sex	
Female	11
Male	4
Practice by deprivation	IMD*
A†	32.9
B	32.0
C	21.3
D	43.7
E	32.0
F	33.7
G	21.7

* The National General Practice Profiles provided the practice deprivation scores in the unit of Index of Multiple Deprivation (IMD) for comparison.^26^

† Anonymised site names for reference.

### Analysis

Interviews were transcribed verbatim and analysed using a thematic approach, to explore barriers, facilitators and impacts of using a risk prediction model to guide AF screening. Interviews were collated, and the initial analysis phase focused on five transcripts selected for their relevance to addressing the question. This initial analysis was followed by analysis of the whole interview dataset (n=15). LS and EH allocated unique identification codes to protect participant identities. Themes were deduced from common topics raised by participants and interview field notes. Key themes were developed through consensus meetings between RN, LS and EH.^18 19^ LS and EH then explored these themes within the wider dataset to establish the veracity of key themes and identify deviant cases, with the themes subsequently refined. These themes were synthesised to understand shared views of the HCPs, aided by reference to social science and health screening literature.^10^

### Ethics

The FIND-AF study was approved by the North West – Greater Manchester South Research Ethics Committee, and the study was approved by the Health Research Authority (Integrated Research Application System project ID: 318197). The nested qualitative study was approved by the School of Medicine Research Ethics Committee at the University of Leeds (MREC 22–028).

### Patient and public involvement

The FIND-AF patient and public involvement (PPI) group have been involved in the FIND-AF programme since its inception. The FIND-AF PPI group has advised on the design, management and delivery of the programme and codesigned the FIND-AF study including participant-facing materials.

## Results

Extracts from the interviews are included in the synthesis of results for reference; the quotations are assigned to the participant identification number (ID 1–15) and practice code (A-G).

Four main themes developed: (1) health implications of AF, (2) positives and negatives of risk prediction in AF screening, (3) strategies to implement a risk prediction model and (4) barriers and facilitators to risk-guided AF screening.

### Health implications of AF

Participants, both clinicians and administrative, were clear in their understanding about the impacts and risks associated with having AF. The majority were specific in their concerns, explicitly mentioning risks of stroke and dying as a direct result of the arrhythmia.

It’s important because I think the risk of stroke, in particular, has quite a big impact on lives. I think as a GP, I've got several examples I can think of over the years of patients who've had a stroke who've turned out to have AF after their stroke diagnosis and that stroke had quite a big impact on the wider family. (ID9_A)

The two most common screening approaches for AF identified from the interviews were opportunistic screening versus symptomatic screening.

We’re either picking people up because of symptoms or opportunistically and therefore there are going to be a lot of people who are… under diagnosed. (ID1_A)

…someone would come in with symptoms, and you’d often then do an ECG… that might show AF or it might be normal. (ID13_B)

Several interviewees expressed an increase in patients making appointments from the advice of their smart watches having detected an arrythmia.

Increasingly, we get people coming in from smart watches. (ID14_G)

Accordingly, all HCPs felt there was a need for a nationally standardised approach.

### Positives and negatives of risk prediction outcomes of AF screening on patient care

The ability of an algorithm to pick up individuals at higher risk of developing AF provoked positive responses.

I think that’s always brilliant. I'm a big advocate of prevention rather than cure. So, I think it’s, I think it would be a really good option. (ID9_A)

The quicker you pick up on AF, the better, because we can put them onto an anticoagulation medication and… it just reduces their risk of stroke. (ID3_E)

If it detects someone with asymptomatic AF then that would be potentially a life saved…so it would have huge implications. (ID2_D)

Some clinicians were cautious about provoking patient anxiety by telling patients that they have been predicted to be at a high risk of developing AF. However, this was framed as the need for careful delivery and patient education of the information, rather than opting to not screen patients because of the concern about causing health anxiety.

I suppose you have to be careful how you word that to patients, because if you word it incorrectly, you're going to panic them… I think the communication with the patient would have to be pretty robust. (ID7_B)

Extra education around it and they would be able to understand the implications and stuff. (ID7_B)

Potential health anxiety was countered by the suggestion that it is more important to be made aware of a threat to your health and be proactive in preventative treatments.

I’d rather know and be on medication that reduces my risk of stroke than not be told. (ID3_E)

Another apprehension raised was the impact of the increase in workload generated by the risk prediction tool. There was an awareness expressed of the finite time and resources available in primary care, and therefore, the importance of appointments and clinicians’ work being directed at helping the greatest number of patients possible. This is explained by one respondent referencing the numbers needed to treat as a measure of evaluating the allocation of resources with clinical outcomes.

Numbers needed to treat thing… I don't think it be detrimental to patients directly, but it could be detrimental to patients if we're using up a lot of appointments that don't have any outcome potentially. (ID1_A)

However, many participants believed that the clinical benefits (promoting earlier management) far outweighed the associated increase in workload. Most believed their workload would only increase at the initial implementation of the new tool and would eventually reduce certain aspects of their work (ie, treating fewer stroke patients).

The benefits far outweigh the … extra workload. (ID2_D)

If you're doing preventative medicine, then you know that’s going to help you in the long run. It’s just that you're probably going to increase your workload to begin with. (ID1_A)

Probably more practice based rather than individual workload. (ID9_A)

### Strategies to implement a risk prediction model

Understanding the most efficient and accessible way to implement risk prediction was a consistent theme throughout the interviews. Among participants, there were two main differing views on the most appropriate method of implementation. One common theme was the potential use of pop-ups to highlight high-risk patients for clinicians. Some participants thought that pop-ups were the most effective way to actively engage clinicians, as the clinician would see the risk as soon as they accessed the patient records.

If it popped up and … you're very keen to be proactive about it would be to get them in with an HA [health assistant] to do a pulse cheque potentially. (ID1_A)

… I prefer a pop up… (ID11_F)

However, it was also suggested lots of pop-ups are irritating for GPs.

Lots of GPS are unhappy about kind of things popping up. (ID2_D)

Therefore, a few participants suggested that a risk prediction model could create a list of all the high-risk patients, which could be either reviewed by a senior clinician or by admin staff:

You need to use the algorithm to generate a work list of patients and then probably target those specifically with a specific appointment. So an invitation to say we've done a search, we've identified you as possibly being risk of this, we'd like you to come down for a manual pulse check. (ID9_A)

…if it was a tool where things were flashing up… I think you would have varying results because some people would ignore it… but if it was a list of work … and you had a named clinician I think that would be a bit more efficient. (ID2_D)

Many HCPs positively signposted the potential role of administrative staff in a risk-guided AF screening pathway. For example, with the pop-up approach, it was suggested that administrative staff could also receive pop-ups, allowing them to book high-risk patients specific appointments to be screened for AF. Alternatively, with the risk list, it was suggested that someone from the administrative team could review the list and then enable them to get patients booked in for extra time with clinicians to be screened for AF.

… you click a box on the system and it basically alerts the diabetes admin team and the patient gets a letter explaining the results and the diabetes admin team… [arrange] them an appointment. (ID13_B)

One participant, who was administrative in their role, suggested a comparison to the National Health Service health check. They suggested using an alert on the patient’s home page, which could allow the admin team to book a patient with a healthcare assistant for a review. If the investigations carried out flagged up any signs of AF, the patient could then be tasked on to a clinician’s task list.

Like a QOF [Quality Outcomes Framework] alert [that] comes up on the whole page to say this person’s eligible for an NHS health check… [they] could have an AF review… with a healthcare assistant. (ID12_A)

After initial screening for AF, there were varying suggestions on whether high-risk patients should undergo annual screening, but support among participants for empowering patients with information about AF, red flags symptoms and self-checking their pulse.

If they’ve got cardiovascular risk factors and hypertension…could be part of their annual screening. (ID7_B)

You could do some education regarding self-checking…symptoms to watch out for palpitations, dizziness. (ID1_A)

### Barriers and facilitators to risk-guided AF screening

A common barrier identified was that if the risk-guided screening was incorporated within routine workflows, then it is likely not to be prioritised given time pressures on HCPs.

The obvious thing that comes to mind because currently we get lots of pop ups that…you know if we're running 20 minutes late, as we often are, unfortunately we do tend to just close them because we just don't have time to action them all. (ID7_B)

By contrast, formal education for both the staff and patients was widely acknowledged as an important facilitator for use in clinical practice. For staff, training on how to use a risk prediction model and reminders of the clinical significance of AF and, thus, stroke reduction, were identified as measures to improve motivation.

I think the main [facilitators] are education for patient and for staff on how to use it and then perhaps what would the follow-up be in 12 months … once it is implemented maybe doing audits of the use of the tool and how effective it’s been. (ID6_B)

Also, many participants felt that patients should be made aware of the health implications of AF while limiting health anxiety. Several participants identified the need for a formal—but simple—protocol for how to follow-up patients calculated as high risk, to ensure equity between practices so individual patients get the correct investigations.

What we need is a rigorous, easy protocol… if it’s not easy… and if it’s got too many steps, nobody’s going do it… it’s got to be simple and easy. (ID11_F)

## Discussion

### Principal findings

We investigated the perceptions of the primary care staff, across a range of occupational roles, at practices participating in the FIND-AF study to the use of a risk prediction model for AF to guide AF screening. This small and select sample of participants showed support for the early detection of AF to reduce the risk of complications, such as stroke. Their accounts were in favour of having a formalised risk-guided approach to detect AF early compared with current practice. The barrier most frequently expressed was the current workload in primary care, though participants perceived that the benefits of risk-guided AF screening outweighed this added burden, and that appropriate use of administrative staff was key to implementation.

### Strengths and limitations of this study

Our study provides novel evidence for the opinions of HCPs on the use of risk to guide AF screening. Importantly, by gathering views from staff in a range of primary care job roles, both patient-facing and administrative, this study contributes insights into how the collaboration of staff can enable the successful implementation of changes to routine care.^20^ Furthermore, by recruiting sites from areas with a range of deprivation, it is more likely that the views expressed by HCPs are influenced by interactions reflective of the entirety of society.

This was a study including HCPs from participating sites in a risk-guided AF screening study and does not address the views of HCPs at practices that chose not to take part. Nevertheless, focusing on HCPs who have engaged has allowed us to understand the information and logistical needs for a risk-guided AF screening pathway. The high proportion of women participants is a common occurrence in qualitative studies and may have affected the findings.^11^ Qualitative analysis is inherently subjective as it is influenced by the assumptions, beliefs and biases of the researcher.^8 21^ In this study, the data collection and analysis were led by two researchers not involved in the development of prediction models for incident AF or the wider FIND-AF study (AH and LS), which may have reduced biases due to preconceived ideas about results. Though we were asking primary care HCPs for their views on risk-guided AF screening, as a possible care setting for AF screening to be conducted, it is possible that practising HCPs will not be familiar with the most contemporary research in this area, and previous research has suggested that up-to-date information on AF screening should be provided to primary care HCPs ahead of an AF screening pathway implementation.^13^

### Findings in relation to other studies

Our results build on previous studies which demonstrated that HCPs and regulators view primary care as the most appropriate location for AF screening and software systems in primary care as a potential digital and transformative route to identify suitable patients for AF screening.^8^ We also mirror general trends of HCPs encountering patients attending with AF diagnoses from privately owned wearable devices,^8 22^ and that this partly influences HCP attitudes that a formal, controlled approach to AF detection is required.

Similar to previous studies that have summarised views of patients,^10 11^ HCPs^12^ and regulators,^8^ we found support for AF screening, which is disproportionate to the level of the current evidence base for the relative risks and benefits of AF screening.^23^,^25^While previous reports have suggested that prompts on the primary care computer system may be an effective way of facilitating AF screening,^13^ here we found more conflicting views. Furthermore, previous studies have shown that incorporating AF screening within a standard primary care consultation is viewed as burdensome and inefficient.^14^ Our study demonstrates novel insights into the role that administrative staff could play in organising a list of patients for AF screening and administering a pathway that runs in parallel to standard consultations. Previous reports have also shown that HCPs and regulators feel that prolonged ECG monitoring for a number of weeks is the most effective approach to detect new AF cases, but that this approach is too expensive within existing resources in healthcare settings.^8^ This may explain the support we observed for the use of a risk prediction tool to guide AF screening. Even though it was recognised that informing patients of a ‘risk’ status may engender health anxiety, previous reports have suggested that patients may draw binary concepts that it is better to be aware of a problem early rather than late, when they perceive outcomes to be poorer.^10^

### Implications for practice and research

Our study highlighted the importance to HCPs of a standardised implementation procedure for a risk prediction model to guide AF screening. As observed before, time was the main barrier to implementation,^12^ but our participants felt this could be mitigated by the administrative team leading on the calculation of the risk score and organisation of appointments/testing. In particular, participants were used to having patients booked in for health checks, and it was seen that an AF screening pathway could be implemented in a similar way.

In addition to the implementation of AF screening, there was concern from HCPs as to what the longitudinal follow-up of patients at ‘high’ risk should be. There was variation in how this may be organised, but a notable finding was the use of the classification to empower patients to be aware of signs, symptoms and even consider self-checks. However, previous studies have demonstrated that patients, even those participating in AF screening studies, have an incomplete understanding of AF and often conflate it with other heart diseases or even high blood pressure.^11^ Thus, before taking this approach there is a need to provide clear and concise information about AF and to check patient understanding, which could be formalised through checklists or decision aids.^11^ Historically, there have been sex-based and age-based discrepancies in how HCPs have counselled about cardiovascular risk or instituted risk modification, as well as how patients perceive their risk before and after consultations.^26 27^ Furthermore, there is variation in communication of risk between specialists and general practitioners, and there is the potential for HCPs to manipulate risk communication and treatment choices if they take a more paternalistic approach.^26^ Further research is required into how risk is communicated and received to determine appropriate personalised approaches across primary care settings.

## Conclusion

This qualitative study of clinical and non-clinical HCPs in primary care taking part in a risk-guided AF screening study demonstrates support for this approach. Practicalities of the impact on HCP working, how a pathway should be organised and how to communicate risk information to patients emerged as key barriers and facilitators for implementation in clinical practice.

## supplementary material

10.1136/bmjopen-2024-091675 online supplemental file 1

## Data Availability

Data are available upon reasonable request.
