# Supplementary material for: Perceptions of healthcare professionals on the use of a risk prediction model to inform atrial fibrillation screening: qualitative interview study in English primary care
Source: BMJ Open. 2025 Feb 5;15(2):e091675. doi: 10.1136/bmjopen-2024-091675 (PMC11800197; doi:10.1136/bmjopen-2024-091675)
Supplement: online supplemental file 1 [file bmjopen-15-2-s001.docx]

**Supplementary Appendix**

The views of healthcare professionals on the use of a risk prediction model to inform atrial fibrillation screening: Qualitative interview study in English primary care

Ellen Hamilton*, Lydia Shone*, Catherine Reynolds, Jianhua Wu, Ramesh Nadarajah*, Chris P Gale*

[Appendix 1: Semi-Structured Interview Guide 2](#_Toc172722355)

## Appendix 1: Semi-Structured Interview Guide

Introduction and Consent Process

Researcher(s) to introduce themselves prior to commencing the interview and check the participants understanding of atrial fibrillation, and the FIND-AF study. Informed consent and permission to audio record the interview obtained from the participant.

Interview close

Thank participants for taking part in the study. Remind them that their responses will remain confidential. Ask participants if they have any questions. Let participants know they can contact the research team at any time using the details on the information leaflet, including if they have any further questions or wish to remove their responses from the study.

| Professional Group: | Questions: | Prompts: |
| --- | --- | --- |
| Questions for all participants | Do you think that detecting people who are at a higher risk of AF is important? |  |
|  | What is your understanding of how AF is detected in clinical practice? | Are you aware of any current methods for screening for AF?  Are you aware of any support tools to help detect patients at high risk of AF? |
|  | Which type of staff will be the main individuals who interact with the results of our screening tool (FIND-AF)? | Do you think it will be HCPs (doctors/nurses) or administrative staff who can alter clinical workflows? |

| Professional Group: | Questions: | Prompts: |
| --- | --- | --- |
| Questions for Healthcare professionals (GPs, Nurses/Nurse practitioners, HCAs | As a healthcare professional, do you feel as though you benefit from the information obtained by the FIND-AF algorithm? | If you had FIND-AF available, would you use it?  If yes, why?  If no, why? |
|  | Do you think implementing the FIND-AF tool would improve patient care? | Patient outcomes?    Can you think of any reasons why it could negatively impact patient care? |
|  | What would you do when a patient is identified as high risk of developing AF? | How would you follow up with this patient?  Investigations, explanation.    What process of referral seems feasible?  e.g., speciality, timeframe.    Who would do the initial follow up? |
|  | How would a new decision support tool for AF impact your job? | Do you think initially it would increase your workload, if so, how?  e.g., learning how to use a new algorithm, time taken to interpret results, time taken to refer patients.  Prescribing anticoagulation.    Do you think in the long-term it would decrease your workload, if so, how?  e.g., reducing incidence of stroke and associated co-morbidities. |
|  | What do you think the barriers are, if any, in implementing FIND-AF into GP practices? | Can you think in terms of individual and nationwide barriers?  Healthcare professional barriers.  Structural barriers.    Can you think of any measures that would make using FIND-AF easier? |

| Professional Group: | Questions: | Prompts: |
| --- | --- | --- |
| Healthcare Regulator (Practice manager, Receptionist) | How would implementing a new algorithm into your practice impact your work? | How would organising/facilitating the training for HCPs to use the tool affect you?    Are you aware of any system related barriers for implementation? |
|  | Are you concerned about any additional incurred costs from implementing a new decision support algorithm into your practice? | If so, what causes of additional costs can you think of?  Costs for additional referrals?  Cost for the algorithm?  Cost for training? |

## 
