# Supplementary material for: Perceptions of healthcare professionals on the use of a risk prediction model to inform atrial fibrillation screening: qualitative interview study in English primary care
Source: BMJ Open. 2025 Feb 5;15(2):e091675. doi: 10.1136/bmjopen-2024-091675 (PMC11800197; doi:10.1136/bmjopen-2024-091675)
Supplement: Supplementary file 2 [file bmjopen-2024-091675.reviewer_comments.pdf]

PEER REVIEW HISTORY

BMJ Open publishes all reviews undertaken for accepted manuscripts. Reviewers are asked to complete a checklist review form and are provided with free text boxes to elaborate on their assessment. These free text comments are reproduced below.

ARTICLE DETAILS

Title (Provisional)

Perceptions of healthcare professionals on the use of a risk prediction model to inform atrial fibrillation screening: Qualitative interview study in English primary care

Authors

Hamilton, Ellen; Shone, Lydia; Reynolds, Catherine; Wu, Jianhua; Nadarajah, Ramesh; Gale, Chris

---

VERSION 1 - REVIEW

**Reviewer**	**1**
**Name**	**Jones, Nicholas**
**Affiliation**	**University of Oxford, Primary Care Health Sciences**
**Date**	**28-Oct-2024**
**COI**	**None**

Thank you for the opportunity to read this qualitative study exploring the perceptions of primary care health professionals with regard to AF screening. There is significant interest in the topic area at a research and policy level, which make the results of interest. I applaud the authors efforts in interviewing a range of practice staff to think about how the whole practice might adopt such a primary care intervention. I found the paper clearly presented and well written.

- I found some of the results fairly cursory e.g. the fact smart watches are being increasingly used by patients with the single quote 'Increasingly, we get people coming in from smart watches'. Similarly, there is a section on workload reporting the participants recognised longer-term work load reductions might offset up front additional work that would be interesting to expand. By contrast, the 'implications for practice section is very long - around 1/6 of the whole paper. Would the authors consider developing some of the results further and cutting some of the Discussion if needed for the word count?

My concerns:

- The interviews were typically short (mean = 13mins) and this is a small and select sample of participants, e.g. all from practices already taking part in the FIND-AF study. I wonder

whether the general report for AF screening reflects this and I would recommend against trying to make generalising statements from this type of sample (e.g. "participants showed broad support for the early detection of AF"), which some readers may interpret as suggesting their is broad support among primary care clinicians for early detection of AF.

- Thank you for including the interview questions in your supplemental material. However, I worry some of these might have been phrased as leading questions. For example, asking "Do you think that detecting people who are at higher risk of AF is important?" would be very likely to provoke an answer of 'Yes'! More open questions might have helped probe participants around the relative merits of early AF detection compared to other interventions we are asked to implement in primary care. How the questions were framed seems fairly fundamental to me as such leading questions may partially account for headline results such as the support for early detection of AF among participants. This is particularly true given these were one to one interviews where one might expect less discussion anyway as opposed to small group sessions with participants.

- The Introduction states that 'views of primary care HCPs on AF screening... are unknown' but this is not the first qualitative primary care study on this topic (e.g. Callanan A et al. Facilitators and barriers to atrial fibrillation screening in primary care: a qualitative descriptive study of GPs in primary care in the Republic of Ireland. BJGP Open. 2023 Jun 27;7(2):BJGPO.2022.0110. doi: 10.3399/BJGPO.2022, or Uittenbogaart SB et al. Experiences with screening for atrial fibrillation: a qualitative study in general practice. BJGP Open. 2022 Mar 22;6(1):BJGPO.2021.0126. doi: 10.3399/BJGPO.2021.0126). These papers identify similar themes e.g. around additional workload as a barrier to AF screening or the potential for alerts and education as interventions to improve screening initiatives. I think the introduction and discussion would benefit from recognising the similarity of these results to previous research. How novel are the findings in this context?

- Although I agree that it is important asking primary care clinicians their views on AF screening, I wonder if the limitations should acknowledge that the participants are unlikely to be familiar with key research that has already taken place in this field. For example electronic alerts (or pop ups) are suggested as a method for implementation, yet the AURAS-AF study found this had no impact on anticoagulation prescribing among people with AF (see Holt et al 2017 in Stroke: https://doi.org/10.1161/STROKEAHA.116.015468). Im also very surprised to see anyone in primary care suggesting a daily review of a list of high-risk patients - could you add a direct participant quote supporting that! (line 263, page 15).

Reviewer **2**

Name **Romiti, Giulio Francesco**

Affiliation **Sapienza University of Rome, Department of Translational and Precision Medicine**

**Date**	**30-Nov-2024**
**COI**	**None**

In this study, the authors report the results of a qualitative interview study among primary care physicians/workers in England, regarding perceptions of HCP on the use of a risk prediction model to guide AF screening. The authors performed interviews in 15 HCP (including doctors, nurses and other healthcare workers). Main results of the interviews are reported and discussed. Generally, HCPs supported the use of risk prediction to guide AF screening, and recognize the importance of screening for AF; they also highlighted potential barriers to the implementation of screening (increasing workload, time constraints etc.).

The study is interesting and the results are useful as they can be used to design and refine interventions/programme aiming at implementing risk prediction models to guide screening of AF. The paper is well written and results are discussed appropriately.

There are no specific comments on the scientific content; authors should be congratulated for this work.

Minor comments that can be amended at proof stage:

- Line 326, "Principle findings" - perhaps "Principal findings")?

- Line 404, "airds" probably a typo ("decision aids"?)

VERSION 1 - AUTHOR RESPONSE

Reviewer: 1
Dr. Nicholas Jones, University of Oxford
Comments to the Author:
Thank you for the opportunity to read this qualitative study exploring the perceptions of primary care health professionals with regard to AF screening. There is significant interest in the topic area at a research and policy level, which make the results of interest. I applaud the authors efforts in interviewing a range of practice staff to think about how the whole practice might adopt such a primary care intervention. I found the paper clearly presented and well written.

Author reply

We thank the Reviewer for taking the time to review the manuscript and providing helpful suggestions.

- I found some of the results fairly cursory e.g. the fact smart watches are being increasingly used by patients with the single quote 'Increasingly, we get people coming in from smart watches'. Similarly, there is a section on workload reporting the participants recognised longer-term work load reductions might offset up front additional work that would be interesting to expand. By contrast, the 'implications for practice section is very long - around

1/6 of the whole paper. Would the authors consider developing some of the results further and cutting some of the Discussion if needed for the word count?

Author reply

We thank the Reviewer for this suggestion. We have added extra quotes throughout to provide more depth to the Results. We have also shortened the 'Implications for Practice' section of the Discussion.

Manuscript change

The two most common screening approaches for AF identified from the interviews were opportunistic screening vs symptomatic screening.

> "We're either picking people up because of symptoms or opportunistically and therefore there are going to be a lot of people who are… under diagnosed" (ID1_A)
>
> "...someone would come in with symptoms, and you'd often then do an ECG… that might show AF or it might be normal" (ID13_B)

…

The ability of an algorithm to pick up individuals at higher risk of developing AF provoked positive responses.

> "I think that's always brilliant. I'm a big advocate of prevention rather than cure. So, I think it's, I think it would be a really good option." (ID9_A)
>
> "The quicker you pick up on AF, the better, because we can put them onto an anticoagulation medication and… it just reduces their risk of stroke." (ID3_E)
>
> "If it detects someone with asymptomatic AF then that would be potentially a life saved…so it would have huge implications." (ID2_D)

Some clinicians were cautious about provoking patient anxiety by telling patients they have been predicted to be at high risk of developing AF. However, this was framed as the need for careful delivery, and patient education, of the information rather than opting to not screen patients because of concern about causing health anxiety.

> "I suppose you have to be careful how you word that to patients, because if you word it incorrectly, you're going to panic them… I think the communication with the patient would have to be pretty robust." (ID7_B)
>
> "Extra education around it and they would be able to understand the implications and stuff." (ID7_B)

…..

However, many participants believed that the clinical benefits (promoting earlier management) far outweighed the associated increase in workload. Most believed their

workload would only increase at the initial implementation of the new tool and would eventually reduce certain aspects of their work (ie treating fewer stroke patients).

> "The benefits far outweigh the ... extra workload." (ID2_D)

> "If you're doing preventative medicine, then you know that's going to help you in the long run. It's just that you're probably going to increase your workload to begin with." (ID1_A)

> "Probably more practice based rather than individual workload." (ID9_A)

….

Understanding the most efficient and accessible way to implement risk prediction was a consistent theme throughout the interviews. Amongst participants, there were two main differing views on the most appropriate method of implementation. One common theme was the potential use of pop-ups to highlight high-risk patients for clinicians. Some participants thought that pop-ups were the most effective way to actively engage clinicians, as the clinician would see the risk as soon as they accessed the patient records.

> "If it popped up and … you're very keen to be proactive about it would be to get them in with an HA [health assistant] to do a pulse cheque potentially." (ID1_A)

> "... I prefer a pop up…" (ID11_F)

However, it was also suggested lots of pop-ups are irritating for GPs.

> "Lots of GPS are unhappy about kind of things popping up." (ID2_D)

….

Therefore, a few participants suggested that a risk prediction model could create a list of all the high-risk patients, which could be either reviewed by a senior clinician, or by admin staff:

> "You need to use the algorithm to generate a work list of patients and then probably target those specifically with a specific appointment. So an invitation to say we've done a search, we've identified you as possibly being risk of this, we'd like you to come down for a manual pulse check." (ID9_A)

> "...if it was a tool where things were flashing up… I think you would have varying results because some people would ignore it… but if it was a list of work … and you had a named clinician I think that would be a bit more efficient." ID2_D

Alternatively, with the risk-list, it was suggested that someone from the administrative team could review the list and then enable them to get patients booked in for extra time with clinicians to be screened for AF.

" ..you click a box on the system and it basically alerts the diabetes admin team and the patient gets a letter explaining the results and the diabetes admin team… [arrange] them an appointment." ID13_B

My concerns:
- The interviews were typically short (mean = 13mins) and this is a small and select sample of participants, e.g. all from practices already taking part in the FIND-AF study. I wonder whether the general report for AF screening reflects this and I would recommend against trying to make generalising statements from this type of sample (e.g. "participants showed broad support for the early detection of AF"), which some readers may interpret as suggesting their is broad support among primary care clinicians for early detection of AF.

Author reply

We agree with the reviewer that this is a small and select sample of participating healthcare professionals taking part in a risk-guided AF screening study, and should not be take to reflect the general perceptions of primary care healthcare professionals. Therefore we have altered the text to reflect this.

Manuscript change

Abstract
Conclusions
Overall, HCPs participating in the FIND-AF study were supportive of using risk prediction to guide AF screening and willing to take on extra workload to facilitate risk-guided AF screening.

Principal findings
We investigated the perceptions of primary care staff, across a range of occupational roles, at practices participating in the FIND-AF study to the use of a risk prediction model for AF to guide AF screening. This small and select sample of participants showed support for the early detection of AF to reduce the risk of complications such as stroke.

Conclusion
This qualitative study of clinical and non-clinical HCPs in primary care taking part in a risk-guided AF screening study demonstrates support for this approach.

- Thank you for including the interview questions in your supplemental material. However, I worry some of these might have been phrased as leading questions. For example, asking "Do you think that detecting people who are at higher risk of AF is important?" would be very likely to provoke an answer of 'Yes'! More open questions might have helped probe participants around the relative merits of early AF detection compared to other interventions we are asked to implement in primary care. How the questions were framed seems fairly fundamental to me as such leading questions may partially account for headline results such as the support for early detection of AF among participants. This is particularly true given these were one to one interviews where one might expect less discussion anyway as opposed to small group sessions with participants.

Author reply

As the Reviewer has previously noted the interviews were relatively short to enable feasibility of recruiting from all members of a busy primary care team. Aware that there

would be time pressure for participants we were keen to extract their experiences and perceptions in as efficient and granular nature as possible. As part of this approach we developed the interview questions by considering the most appropriate approach across the doxastic to epistemic continuum (https://doi.org/10.1177/1077800418810724). We chose a doxastic approach and considered that a selection of more direct questions was an important component of the interviews as fully open-ended questions can present an insurmountable challenge to extracting similar themes and codes from the interviews. We were also informed by previous qualitative studies on AF screening (http://dx.doi.org/10.1136/bmjopen-2021-051703) and feel our questioning style was not significantly different. We take onboard the comment and will consider incorporating a more open-ended interview style in follow-on work.

- The Introduction states that 'views of primary care HCPs on AF screening... are unknown' but this is not the first qualitative primary care study on this topic (e.g. Callanan A et al. Facilitators and barriers to atrial fibrillation screening in primary care: a qualitative descriptive study of GPs in primary care in the Republic of Ireland. BJGP Open. 2023 Jun 27;7(2):BJGPO.2022.0110. doi: 10.3399/BJGPO.2022, or Uittenbogaart SB et al. Experiences with screening for atrial fibrillation: a qualitative study in general practice. BJGP Open. 2022 Mar 22;6(1):BJGPO.2021.0126. doi: 10.3399/BJGPO.2021.0126). These papers identify similar themes e.g. around additional workload as a barrier to AF screening or the potential for alerts and education as interventions to improve screening initiatives. I think the introduction and discussion would benefit from recognising the similarity of these results to previous research. How novel are the findings in this context?

Author reply

We thank the Reviewer for highlighting these important studies, which we have now incorporated in the manuscript. We incorporate the difference between our study and these, that we include non-patient facing administrative staff, and that our study relates to the use of a risk prediction model to guide AF screening, in the introduction. In the discussion we have also incorporated a paragraph highlighting the novel findings we demonstrate in addition, and in contrast, to what was found in these studies.

Manuscript change

Introduction
The perceptions of general practitioners, nurse practitioners, and healthcare assistants for the facilitators and barriers of AF screening have been explored,[13 14] but not administrative staff, and not specifically related to the implementation of a risk prediction model.

Discussion
Whilst previous reports have suggested that prompts on the primary care computer system may be an effective way of facilitating AF screening,[13] here we found more conflicting views. Furthermore, previous studies have shown that incorporating AF screening within a standard primary care consultation is viewed as burdensome and inefficient.[14] Our study demonstrates novel insights into the role that administrative staff could play in organizing a list of patients for AF screening, and administering a pathway that runs in parallel to standard consultations.

- Although I agree that it is important asking primary care clinicians their views on AF screening, I wonder if the limitations should acknowledge that the participants are unlikely to be familiar with key research that has already taken place in this field. For example electronic alerts (or pop ups) are suggested as a method for implementation, yet the AURAS-AF study found this had no impact on anticoagulation prescribing among people with AF (see Holt et al 2017 in Stroke: https://doi.org/10.1161/STROKEAHA.116.015468). Im also very surprised to see anyone in primary care suggesting a daily review of a list of high-risk patients - could you add a direct participant quote supporting that! (line 263, page 15).

Author reply

We agree with the Reviewer that the support we and other qualitative studies have found for AF screening, not withstanding the biases in ours and other studies, is disproportionate to the evidence base for AF screening from randomised clinical trials. Hence in the discussion we noted: "Similarly to previous studies that have summarized views of patients,[10 11] HCPs,[23] and regulators,[8] we found support for AF screening, which is disproportionate to the level of the current evidence base for the relative risks and benefits of AF screening.[24-26]"

We have now also added to the limitations section.

Manuscript change

Though we were asking primary care HCPs for their views on risk-guided AF screening, as a possible care setting for AF screening to be conducted, it is possible that practising HCPs will not be familiar with the most contemporary research in this area, and previous research has suggested that up-to-date information on AF screening should be provided to primary care HCPs ahead of an AF screening pathway implementation.[13]

Reviewer: 2
Dr. Giulio Francesco Romiti, Sapienza University of Rome
Comments to the Author:
In this study, the authors report the results of a qualitative interview study among primary care physicians/workers in England, regarding perceptions of HCP on the use of a risk prediction model to guide AF screening. The authors performed interviews in 15 HCP (including doctors, nurses and other healthcare workers). Main results of the interviews are reported and discussed. Generally, HCPs supported the use of risk prediction to guide AF screening, and recognize the importance of screening for AF; they also highlighted potential barriers to the implementation of screening (increasing workload, time constraints etc.).

The study is interesting and the results are useful as they can be used to design and refine interventions/programme aiming at implementing risk prediction models to guide screening of AF. The paper is well written and results are discussed appropriately.

There are no specific comments on the scientific content; authors should be congratulated for this work.

Author reply

We thank the Reviewer for their supportive comments, and for taking the time to review our work.

Minor comments that can be amended at proof stage:
- Line 326, "Principle findings" - perhaps "Principal findings")?
- Line 404, "airds" probably a typo ("decision aids"?)

Author reply

We thank the Reviewer for pointing out these errors. We have now amended them.

Manuscript change

Principal findings

…

decision aids

VERSION 2 - REVIEW

**Reviewer**	**1**
**Name**	**Jones, Nicholas**
**Affiliation**	**University of Oxford, Primary Care Health Sciences**
**Date**	**06-Jan-2025**

COI

Thanks for updating the paper. I have no further suggestions and would be happy to see this published.
